# The relationship between psychosocial factors, self-care, and blood sugar in an Appalachian population

**DOI:** 10.13023/jah.0403.01

**Published:** 2023-01-01

**Authors:** Brittany L. Smalls, Tofial Azam, Madeline Dunfee, Philip M. Westgate, Susan C. Westneat, Nancy Schoenberg

**Affiliations:** University of Kentucky, brittany.smalls@uky.edu; University of Kentucky; University of Kentucky; University of Kentucky

**Keywords:** Appalachia, psychosocial, diabetes, self-care

## Abstract

**Introduction:**

Appalachian residents are more likely than other populations to have Type 2 Diabetes Mellitus (T2DM) and to experience more severe complications from the disease, including excess and premature mortality.

**Methods:**

This study examines health alongside sociodemographic factors, psychosocial factors (including knowledge, empowerment, social support/function, religiosity, distress), and perceived problems in diabetes management that may influence self-care and HbA1c among vulnerable rural residents. A survey of a community–based sample of 356 adults with diagnosed diabetes or HbA1c > 6.5 was conducted in six counties in Appalachian Kentucky.

**Results:**

Findings suggest that neither religiosity nor social support/function mediate/moderate the relationship between psychosocial factors and dependent variables (problem areas in diabetes, T2DM self-care or HbA1c). Results also suggest that distress is a predictor of problem areas in diabetes, and both distress and empowerment are predictors of T2DM self-care.

**Implications:**

This study addresses the gap in the literature concerning the influence of psychosocial factors on problem areas in diabetes, T2DM self-care and HbA1c among vulnerable rural residents, as well as the potential mediating/modifying effects of religiosity and social function/support. Future research is needed to inform strategies for identifying and addressing distress among vulnerable populations burdened by T2DM, including Appalachian adults.

## INTRODUCTION

With nearly 10% of the U.S. population (29 million Americans) having been diagnosed with Type 2 Diabetes Mellitus (T2DM) and another 86 million considered “prediabetic,” T2DM can be considered an epidemic.^1^–^3^ As the leading cause of new cases of blindness, kidney disease, and amputations, T2DM threatens the health of millions of Americans. T2DM also takes a financial toll on the nation, with over $174 billion in associated costs.^4^ Of particular concern are those who are disproportionately affected by T2DM, including members of lower socioeconomic status (SES), minority, and rural populations.^3^ Rural Appalachian residents exemplify these vulnerable populations, with rates of diabetes 46% higher than national averages^3^,^5^ and affecting up to 23% of the community in some Appalachian regions.^6^

Numerous determinants have been proposed to explain the high rates of T2DM in rural Appalachia, including system-, community-, practitioner-, and patient/individual-level factors. These determinants, which correspond with the Chronic Care Model, include resources, policies, delivery systems, clinical information systems, decision support, and psychosocial factors (e.g., knowledge, distress, and empowerment). These psychosocial factors have been shown to influence behaviors, including self-care and formal medical management.^7^,^8^ Less well known is how psychosocial factors affect actual average blood glucose (HbA1c) levels in heavily burdened rural populations.

Existing evidence suggests that Appalachian residents are at elevated risk of and from T2DM. Established T2DM risk factors, including elevated body mass index and older age, are common across Appalachia; for example, 31% of Appalachian adults are obese,^9^ and the median age is increasing across many Appalachian subregions.^11^ Furthermore, the incidence of T2DM-related mortality is 11% higher across Appalachia than on average for the nation.^9^

Extensive research suggests that psychosocial factors may play a role in determining the self-care behaviors that ultimately influence HbA1c and diabetes outcomes. Greater knowledge about T2DM and recommended self-care behaviors has been associated with diabetes control and better outcomes.^11^ Diabetes distress—a negative emotional and behavioral response to managing a demanding chronic condition like T2DM—is estimated to occur in nearly half of all adults with diabetes.^12^ Research has demonstrated that even moderate levels of diabetes distress may lead to substandard adherence and elevated HbA1C levels.^13^ Conversely, achieving lower levels of distress through interventions has been shown to improve HbA1C. Diabetes empowerment, understood as the psychosocial self-efficacy of people living with diabetes to manage their condition, has been correlated with optimal self-management.^14^,^15^

Insight into the process or mechanism in which psychosocial factors influence HbA1c and diabetes outcomes is lacking; however, researchers have proposed that two additional psychosocial factors—social support and religiosity—may play a mediating and modifying role. Specifically, social support has been shown to moderate the effects of stress and depression on T2DM self-care, which ultimately influences HbA1c. Likewise, religiosity has been shown to moderate the effect of distress on T2DM self-care. Additional research has demonstrated a positive association between religiosity and HbA1c and a negative association between spirituality and HbA1c.^16^,^17^ Despite their greater vulnerability, rural residents have rarely been the focus of research on the influence of psychosocial factors on self-care and, ultimately, HbA1c. This article aims to fill this gap.

## METHODS

This paper reports the results of baseline cross-sectional data collected as part of the ongoing study “Community to Clinic Navigation to Improve Diabetes Outcomes” (R01 DK112136, PI: Schoenberg). The study employs the Chronic Care Model^18^ to better understand and address obstacles to the major determinants of diabetes outcomes. The ongoing intervention addresses both patient activation and clinical support. However, the current study focuses on one component of the Chronic Care model: psychosocial factors (e.g., knowledge, empowerment, self-efficacy, and distress). This article presents an analysis of the relationship between psychosocial factors, self-care and HbA1c from data collected during the baseline interview. All research protocols were approved by the University of Kentucky’s Institutional Review Board (#14-0314-P6H).

### Setting

Participants were recruited from six Appalachian counties classified by the Appalachian Regional Commission as economically distressed, given their high rates of unemployment, poverty, and low income.^19^,^20^ These counties were selected due to a variety of factors: their suboptimal resources and health profiles; our project’s 16-years-long engagement with these counties; and longstanding partnership with local community organizations (e.g., churches, health care facilities, and community centers).^21^

### Recruitment

Participants were recruited through local community organizations, most frequently churches. After contacting a community organization to determine interest in participating in the project, project staff met with a minister or organization director to determine the special needs or considerations of their group. Working within those parameters, researchers then conducted an information session about the project at their sites and invited potentially eligible and interested individuals to chat following the presentation. During that discussion, administered informed consent documents were administered and eligibility assessed. If an individual was deemed eligible, staff took contact information and arranged for a baseline interview within one to two weeks.

### Participants

Eligible participants were aged 18 years or older, resided in Appalachia, had no plans to relocate out of the area in the next 18 months, had the capacity to participate (i.e., no major psychosocial impairment, as assessed by the Montreal Psychosocial Assessment [MoCA] screener), and received a diagnosis of T2DM and/or HbA1c levels of at least 6.5%. Eligible participants were asked to sign an approved informed consent form. Those who completed the written consent form were then subject to pre-enrollment screenings to verify their HbA1c levels were consistent with a diagnosis of T2DM. Given the high prevalence of undetected T2DM in Appalachian communities, interested individuals with no known T2DM diagnosis with an elevated risk of T2DM—determined by a score of ≥ 2 on the American Diabetes Association Risk Test^22^—were offered HbA1c screening. Also, given close-knit rural communities and the likelihood that household members tend to attend church or community centers together, more than one member of a household could be eligible to participate.

### Study Measures

Existing and validated measures were employed if possible and instruments pilot tested prior to initiating the baseline interview.^23^,^24^ A pilot test was necessary because of the limited use of these instruments among the target population.

#### Independent Variables

**Knowledge:** The Michigan Diabetes Research and Training Center’s Revised Diabetes Knowledge Test (DKT2) was administered to assess general knowledge of diabetes and diabetes self-care among those who do not use insulin (the first 14 items) as well as those who use insulin (the final 9 items). Reliability has been reported for non-insulin users (.77) and insulin users (.84).^25^

**Empowerment/Self-efficacy:** The Diabetes Empowerment Scale (DES-SF) was used to assess self-efficacy.^26^ This scale contains eight items, including self-assessed capacity to obtain social support and to manage stress, measured through a Likert scale. The reliability and validity have been well established.^27^,^28^

**Distress:** The 17-item Diabetes Distress Scale (DDS)^29^ focuses on four domains of distress: emotional burden, regimen distress, interpersonal distress, and physician distress. The DDS has been used globally to measure diabetes-related emotional distress and as an instrument in intervention studies. Internal reliability was established (0.87) and validity coefficients indicated significant linkages to the CESD (*p* < 0.001).

#### Potential Mediator/Moderators

**Social Support/Function:** To assess social function and activities, researchers administered the PROMIS® (Patient-Reported Outcomes Measurement Information System)–SF scale, Version 2.0, which has subscales for companionship, informational support, social roles and activities, and emotional support, among other domains.^30^ The instrument has demonstrated cross-cultural validity and reliability, irrespective of disease.^31^

**Religiosity:** To assess religiosity, two questions on a Likert scale were asked. These questions focused on the frequency of attendance at formal religious organizations as well as the extent of time engaged in spiritual and religious practice. In rural environments, religious institutions frequently exert an influence on behavior; thus, these questions were administered in all studies.^32^

#### Clinical Outcome

**Hemoglobin HBA1C (HbA1c):** The primary outcome, HbA1c, was measured with a Bayer DCA 2000+ Analyzer,^33^ which has a test coefficient of variation of < 5% (consistent with requirements of the National Diabetes Data Group).

#### Dependent Variables

**Distress:** We administered the 20-item Problem Areas in Diabetes (PAID) scale,^34^ to assess the degree to which diabetes management and/or feelings about diabetes are problematic to participants. The PAID scale has been psychometrically validated among a diverse array of populations.^35^–^37^

**Diabetes self-care:** The Summary of Diabetes Self-Care Activities (SDSCA) measure was administered to assess key self-care domains including general and specialized diet, exercise, blood sugar testing, and footcare. The SDSCA tends to be highly correlated with other measures of diet and exercise, and it has been employed in a variety of settings with validity and reliability. The self-care scores were averaged to create an overall measure of self-care.^38^

#### Sociodemographic Variables (Covariates)

Data were collected on standard sociodemographic factors known to influence health behavior and outcomes: race, sex, age, marital status, employment status, self-perceived financial status, actual income, education, insurance coverage, household composition, and a comprehensive series of questions on health status. Health status questions included the MoCA (cognitive screener),^39^ the Center for Epidemiological Studies Depression (CESD) Scale,^40^ and the Medical Outcomes Study Health Status Short-Form Survey (SF-36).^41^,^42^

### Data Collection

Data were collected by community interviewers, all of whom had completed the CITI Program and extensive data collection training. Although the data collected were not considered by participants or staff to include intrusive questions, a need for confidentiality was still emphasized. Interviews were conducted in participants’ homes, at the project office, or in another community location. To allay concerns about limited literacy, the interviewers read all questions to participants. Data were collected on iPads using the REDCap mobile app to accommodate situations in which there was no WiFi or cellular connection; in these instances, data were later exported back to the secure REDCap server at the University of Kentucky. A data manager then checked each data entry for completeness and merged biometric data using unique participant ID numbers to create a comprehensive dataset.

**Figure 1:**
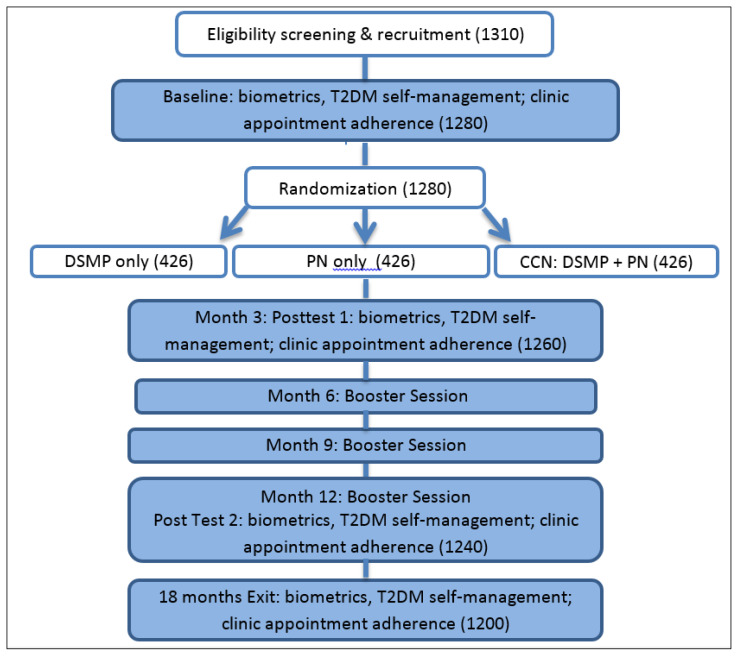
CNN Intervention Project flow, assessments, participant assignment, and outcomes

Interviews ranged from 45 to 80 minutes in length. Upon completion of the baseline interviews, all participants received an honorarium of $35, a standard payment in this region. As shown in Fig. 1, within several weeks, the participant was randomized to an early or delayed intervention arm.

### Statistical Analyses

Regression models assessed mediation and moderation. Multilevel linear mixed-effect models and GEE-type logistic regression models were fit for both continuous outcomes (HbA1c, problem areas in diabetes, self-care, and social support) and binary outcomes (religiosity). Within these mixed models, random site effects and random household effects within sites were used to account for the possibility of multiple levels of clustering due to study design. Because of the high prevalence of religiosity, accounting for such clustering in the GEE-type logistic regression models often results in non-convergence, and therefore a working independence structure with Kauermann and Carroll^43^ bias-corrected standard errors was used to ensure valid inference. To obtain standardized beta coefficients, continuous outcome and predictor variables were centered and standardized. Regression models adjusted for demographic variables (age, sex, marital status, education, employment, financial status, insurance, smoking status, and health conditions).

The results below are based on the approach of Baron and Kenny^44^ for mediation, and also address moderation. Tables 2–4 provide results corresponding to HbA1c, problem areas in diabetes, and self-care, respectively. For each of these dependent variables, either distress, empowerment, or knowledge is used as the independent variable of interest, and religiosity and social support are the potential mediators. In the first step, the independent variable was used to predict the given outcome. In the second step, the independent variable was used in two separate models predicting religiosity and social support/function. The final model used to assess mediation incorporates the independent variable, religiosity, and social support as predictors of the given outcome. Finally, to assess effect moderation, regression models add two covariates that correspond to the interactions of the given independent variable with religiosity and social support.

All tests were two sided. Statistical significance is defined as *p* < 0.05. Analyses are conducted in SAS version 9.4 (SAS Institute, Cary NC).

## RESULTS

Table 1 displays sample characteristics (N=356) used for analysis. The average age of participants was 64.2 years (± 10.6). Most of the sample population were white (98%), female (64.6%), married (58.4%), insured (98%), and nonsmoking (89.6%), with an average HbA1c of 7.7% (± 1.7). Over two-thirds of the sample population had some postsecondary education, and the majority were not employed, either being disabled (20.5%) or retired (42.1%). Participants most frequently reported having no depressive symptoms (69.4%), at least two chronic conditions (65.4%), and being religious (86%). Participants also reported average scores for self-care practices and problem areas in diabetes as 17.1 (± 6.3; range 4.3–33.6) and 7.7 (±1.7; range 5.3 – 14.0), respectively.

### Model 1: Mediation /Moderation Effects of Religiosity and Social Function/Support on HbA1c

The adjusted mediation effects of religiosity and social function/support on HbA1c showed that there were no significant predictors of HbA1c (Table 2). Similarly, when evaluating an independent variable along with religiosity and social function/support to predict HbA1c, no statistically significant predictors were found. However, there was an independent relationship between distress as a predictor of religiosity, indicating that greater distress is associated with a lower likelihood of being religious (OR = 0.454, 95% CI: 0.287–0.717, *p* <0.001). None of the independent variables were significant predictors of social function/support. As a result of this analysis, religiosity and social function/support were not found to be statistically significant predictors of HbA1c.

### Model 2: Mediation/moderation Effects of Religiosity and Social Function/Support on Problem Areas in Diabetes

The results show that distress is a significant predictor of problem areas in diabetes (0.601, se = 0.053, *p* < 0.001; Table 3). When assessing if religiosity and social function/support predict these problem areas, the study found a significant independent association with distress (0.606, se = 0.056, *p* < 0.001). In addition, religiosity had a significant negative association with problem areas in diabetes when either empowerment or knowledge were the independent variables. However, in the adjusted model, the association is negligible (0.014, se = 0.143, *p* = 0.922) when distress is included in the model. Ultimately, neither religiosity nor social support appear to mediate any relationships between distress and problem areas in diabetes.

### Model 3: Mediation /Moderation Effects of Religiosity and Social Function/Support on Self-Care

The adjusted mediation effects of religiosity and social support/function on self-care showed that distress is a significant predictor (−0.398, se = 0.061, *p* < 0.001; Table 4). When including religiosity and social support/function in the model to predict self-care, the study found a significant association with distress (−0.395, se = 0.063, *p* < 0.001). Similarly, the adjusted mediation effects of religiosity and social support/function on self-care showed that empowerment is a statistically significant predictor (0.216, se = 0.057, *p* < 0.001; Table 4). When including religiosity and social function /support in the model to predict self-care, there was a significant association with empowerment (0.211, se = 0.057, <0.001). Overall, neither religiosity nor social function/support were found to be statistically significant mediators.

## DISCUSSION

These baseline data showed that, on average, adults with diabetes or with an HbA1c level ≥ 6.5% reported engaging in a moderate level of self-care activities and experienced several problem areas associated with diabetes. Additionally, few people reported depressive symptoms; conversely, most reported some form of religiosity. Key findings from the mediation analysis indicate that neither religiosity nor social function/support mediate the relationship between the independent variables (knowledge, distress, and empowerment/self-efficacy) and dependent variables (self-care and HbA1c). However, there were other statistically significant relationships that warrant further discussion.

The findings show that distress had a predictive relationship with religiosity: high distress was associated with low religiosity. This finding is consistent with the literature that supports the role of religion and spirituality in influencing chronic disease outcomes, perhaps related to lower levels of disease-related stress.^45^,^46^ Such a finding may prove particularly salient to residents of Appalachia, who tend to be religious and have close connection with faith-based organizations. Indeed, some researchers have proposed that religiosity could be a better proxy measure for social support for residents of Appalachia than validated measures for social support used in the general population. This speculation is supported by literature that shows that high social support among residents of Appalachia do not improve T2DM self-care activities and outcomes, as seen in the general population.^47^ Furthermore, contrary to findings in the general population, these results suggest that social support is not utilized or internalized among residents of Appalachia regarding health and wellbeing.

The data also show that distress has an independent relationship with problem areas in diabetes, religiosity, and social function/support; and religiosity appears to function as a covariate when assessing the relationship between empowerment and knowledge. The independent relationship of distress indicates the importance of identifying and addressing distress among those diagnosed with T2DM, particularly those living in Appalachia. Early detection of distress may mitigate its pervasive effect on personal factors (e.g., religiosity and social function/support) and outcomes of interest (e.g., problem areas in diabetes).

Finally, distress and empowerment had a significant association with religiosity and social function/support as related to self-care. While religiosity and social function/support did not meet the conditions for being mediators, they do affect the relationship between psychosocial factors (e.g., distress and empowerment) and self-care activities. This relationship previously has been noted in the literature.^48^,^49^ However, the mechanism by which distress and empowerment manifest in residents of Appalachia living with T2DM may differ due to social determinants that were not assessed in this analysis, including barriers to clinic care and access to resources that are essential to perform self-care activities, including healthy food outlets and physical-activity resources.

Though this study has provided interesting findings, we also acknowledge its limitations. First, the study sample is limited to a relatively homogenous population of residents in Appalachia; results may not be generalizable to all rural residents or even to other populations living with T2DM. Second, most of those included in the analysis were recruited from churches, which likely provided an oversampling of those who participate in religious activities. Additionally, all surveys were self-reported and not independently verified, leading to possible recall and other bias. Finally, since this study comprised a secondary analysis, key variables with influence over outcomes may have been overlooked.

Four key implications emerge from this study: First, researchers should rethink the role of social function/support and its importance (or lack thereof) for certain populations when it comes to chronic disease management (e.g., diabetes in Appalachian Kentucky). Whether due to a measurement effect (for example, a high ceiling for social function/support) or a negative influence of socially significant others, social function/support did not moderate any of the relationships examined. Second, consistent with existing research, the significant, independent inverse relationship between distress and both religiosity and problem areas may suggest that religiosity is a coping mechanism. Third, since more problem areas indicated higher distress, research should focus on building problem-solving skills to reduce distress. Since religiosity had a significant relationship with distress and problem areas, future research should explore the possibility of a mediating/moderating relationship. Finally, contrary to our expectations, no significant relationship existed between HbA1c and the outcome variables. Future mediation analyses should include self-care and HbA1c to determine if these outcomes can be affected by religiosity or social function/support.

**SUMMARY BOX**

**What is already known about this topic?**

Appalachian residents are more likely than other populations to have Type 2 Diabetes Mellitus (T2DM) and to experience more severe complications from the disease, including excess and premature mortality.

**What is added by this report?**

This study examines health alongside sociodemographic factors, psychosocial factors (including knowledge, empowerment, social support/function, religiosity, distress), and perceived problems in diabetes management that may influence self-care and HbA1c among vulnerable rural residents. Findings suggest that neither religiosity nor social support/function mediate/moderate the relationship between psychosocial factors and dependent variables (problem areas in diabetes, T2DM self-care or HbA1c). Results also suggest that distress is a predictor of problem areas in diabetes, and both distress and empowerment are predictors of T2DM self-care.

**What are the implications for future research?**

Researchers should rethink the role of social function/support and its importance (or lack thereof) for certain populations when it comes to chronic disease management. Even more specifically, future mediation analyses should include self-care and HbA1c to determine if these outcomes can be affected by religiosity or social function/support.

## Figures and Tables

**Table 1:** Characteristics of study participants

	Mean ± SD or N (%)	Range	Missing (*n*)

**Demographic Information**			

**Age**	64.2 ± 10.6	21.4 – 88.9	3

**Sex**			0
Men	126 (35.4%)		
Women	230 (64.6%)		

**Race/Ethnicity**			0
White	349 (98.0%)		
African American	7 (2.0%)		

**Marital Status**			4
Married	208 (58.4%)		
Divorced	55 (15.4%)		
Never Married	21 (5.9%)		
Widowed	68 (19.1%)		

**Education Level**			0
High School/GED	115 (32.3%)		
Associate degree	43 (12.1%)		
Bachelor’s degree	24 (6.7%)		
Some College	61 (17.1%)		
Graduate/Professional degree	113 (31.7%)		

**Employment Status**			0
Full-time	63 (17.7%)		
Disability	73 (20.5%)		
Homemaker	49 (13.8%)		
Part-time	12 (3.4%)		
Retired	150 (42.1%)		
Unemployed	9 (2.5%)		

**Financial Status**			8
Just enough to get by	154 (43.3%)		
More than you need to live well	91 (25.6%)		
Sometimes struggle to make ends meet	103 (28.9%)		

**Insurance Status**			0
Insured	349 (98.0%)		
Uninsured	7 (2.0%)		

**Independent Variables**			

**Health Status**	47.0 ± 21.6	0.0 – 95.0	0

**Stress**	21.8 ± 9.3	0.0 – 47.0	37

**Distress**	28.3 ± 12.2	17.0 – 83.0	22

**Empowerment**	31.7 ± 7.0	8.0 – 40.0	10

**Social Support**	114.7 ± 17.0	51.0 – 150.0	2

**Knowledge**	15.3 ± 3.9	1.0 – 23.0	0

**Depressive Symptoms**			10
Yes	90 (25.3%)		
No	247 (69.4%)		

**Religiosity**			1
Yes	306(86.0%)		
No	49 (13.8%)		

**Smoking Status**			0
Yes	37 (10.4%)		
No	319 (89.6%)		

**Health Conditions**			15
0	17 (4.8%)		
1	72 (20.2%)		
2	161 (45.2%)		
3	58 (16.3%)		
4	24 (6.7%)		
5	7 (2.0%)		
6	2 (0.6%)		

**Dependent Variables**			

**HbA1c**	7.7 ± 1.7		28

**Problem Areas in Diabetes**	7.7 ± 1.7		13

**Diabetes Self Care Total**	17.1 ± 6.3	4.3 – 33.6	1
General Diet	3.12 ± 2.42	0.0 – 9.0	0
Special Diet	3.35 ± 1.58	0.0 – 7.0	0
Exercise	2.02 ± 2.26	0.0 – 7.0	1
Blood Sugar Testing	3.94 ± 2.74	0.0 – 7.0	0
Foot Care	4.70 ± 1.39	0.0 – 7.0	0

**Table 2:** Multilevel regression analysis for the mediation effects of religiosity and social support on predicting HbA1c adjusted for sociodemographic information

	Step 1*		Step 2†				Step 3§	
Dependent Variable	HbA1c		Religiosity		Social Support		HbA1c	
Predictor(s)	Estimate (SE)	*p*-value	Estimate (SE)	*p*-value	Estimate (SE)	*p*-value	Estimate (SE)	*p*-value
**Distress**								
Distress	0.077 (0.063)	0.221	**−0.790** (**0.232)**	**<0.001**	−0.083 (0.062)	0.186	0.070 (0.065)	0.283
Religiosity							−0.026 (0.166)	0.875
Social Support							−0.031 (0.059)	0.283
**Empowerment**								
Empowerment	0.058 (0.059)	0.330	0.215 (0.204)	0.295	0.089 (0.056)	0.114	0.070 (0.60)	0.242
Religiosity							−0.055 (0.167)	0.740
Social Support							−0.062 (0.60)	0.304
**Knowledge**								
Knowledge	0.013 (0.064)	0.844	0.080 (0.244)	0.743	0.026 (0.061)	0.667	0.015 (0.064)	0.813
Religiosity							−0.049 (0.163)	0.762
Social Support							−0.058 (0.059)	0.322

NOTES:

* Step 1: For the given independent variable, an adjusted multilevel linear mixed effects regression model is fit to predict baseline HBA1C.

† Step 2: For the given independent variable, an adjusted GEE-type logistic regression model is fit to predict religiosity, and an adjusted multilevel linear mixed effects regression model is fit to predict social support. Results from the GEE-type logistic regression models are presented in terms of log odds.

§ Step 3: An adjusted multilevel linear mixed effects models is fit incorporating the given independent variable, religiosity, and social support as predictors of baseline HBA1C.

All models adjust for demographic variables.

**Table 3:** Multilevel regression analysis for the mediation effects of religiosity and social support on predicting problem area in diabetes adjusted for sociodemographic information

	Step 1*		Step 2†				Step 3§	
Dependent Variable	Problem areas in diabetes		Religiosity		Social Support		Problem areas in diabetes	
Predictor(s)	Estimate (SE)	*p*-value	Estimate (SE)	*p*-value	Estimate (SE)	*p*-value	Estimate (SE)	*p*-value
**Distress**								
Distress	**0.601** (**0.053)**	**<0.001**	**−0.790** (**0.232)**	**<0.001**	−0.083 (0.062)	0.186	**0.606** (**0.056)**	**<0.001**
Religiosity							0.014 (0.143)	0.922
Social Support							0.051 (0.051)	0.313
**Empowerment**								
Empowerment	−0.070 (0.058)	0.221	0.215 (0.204)	0.295	0.089 (0.056)	0.114	−0.059 (0.058)	0.310
Religiosity							**−0.367** (**0.163)**	**0.025**
Social Support							0.020 (0.060)	0.731
**Knowledge**								
Knowledge	0.079 (0.064)	0.220	0.080 (0.244)	0.743	0.026 (0.061)	0.667	0.081 (0.064)	0.205
Religiosity							**−0.384** (**0.163)**	**0.020**
Social Support							0.035 (0.059)	0.550

NOTES:

* Step 1: For the given independent variable, an adjusted multilevel linear mixed effects regression model is fit to predict problem areas in diabetes.

† Step 2: For the given independent variable, an adjusted GEE-type logistic regression model is fit to predict religiosity, and an adjusted multilevel linear mixed effects regression model is fit to

§ Step 3: An adjusted multilevel linear mixed effects models is fit incorporating the given independent variable, religiosity, and social support as predictors of problem areas in diabetes.

All models adjust for demographic variables.

**Table 4:** Multilevel regression analysis for the mediation effects of religiosity and social support on predicting self-care adjusted for sociodemographic information

	Step 1*		Step 2†				Step 3§	
Dependent Variable	Self-Care		Religiosity		Social Support		Self-Care	
Predictor(s)	Estimate (SE)	*p*-value	Estimate (SE)	*p*-value	Estimate (SE)	*p*-value	Estimate (SE)	*p*-value
**Distress**								
Distress	**−0.398** (**0.061)**	**<0.001**	**−0.790** (**0.232)**	**<0.001**	−0.083 (0.062)	0.186	**−0.395** (**0.063)**	**<0.001**
Religiosity							0.057 (0.165)	0.730
Social Support							−0.053 (0.057)	0.360
**Empowerment**								
Empowerment	**0.216** (**0.057)**	**<0.001**	0.215 (0.204)	0.295	0.089 (0.056)	0.114	**0.211** (**0.057)**	**<0.001**
Religiosity							0.272 (0.164)	0.098
Social Support							−0.060 (0.058)	0.301
**Knowledge**								
Knowledge	0.063 (0.063)	0.322	0.080 (0.244)	0.743	0.026 (0.061)	0.667	0.061 (0.063)	0.338
Religiosity							0.288 (0.165)	0.083
Social Support							−0.043 (0.058)	0.481

NOTES:

* Step 1: For the given independent variable, an adjusted multilevel linear mixed effects regression model is fit to predict self-care.

† Step 2: For the given independent variable, an adjusted GEE-type logistic regression model is fit to predict religiosity, and an adjusted multilevel linear mixed effects regression model is fit to predict social support. Results from the GEE-type logistic regression models are presented in terms of log odds.

§ Step 3: An adjusted multilevel linear mixed effects models was fit incorporating the given independent variable, religiosity, and social support as predictors of self-care.

All models adjust for demographic variables.
